# Autonomous Navigation for Autonomous Underwater Vehicles Based on Information Filters and Active Sensing

**DOI:** 10.3390/s111110958

**Published:** 2011-11-22

**Authors:** Bo He, Hongjin Zhang, Chao Li, Shujing Zhang, Yan Liang, Tianhong Yan

**Affiliations:** 1 School of Information Science and Engineering, Ocean University of China, 238 Songling Road, Qingdao 266100, China; E-Mails: excellentzhj@163.com(H.Z.); lich07@126.com (C.L.); sjzhang365@gmail.com (S.Z.); liangyan1210@126.com(Y.L.); 2 School of Mechanical & Electrical Engineering, China Jiliang University, 258 Xueyuan Street, Xiasha High-Edu Park, Hangzhou 310018, China; 3 State Key Lab of Digital Manufacturing and Equipments Technology, Huazhong University of Science and Technology, Luoyu Road, Wuhan 430074, China

**Keywords:** autonomous underwater vehicle (AUV), extended information filter, localization, navigation, sonar

## Abstract

This paper addresses an autonomous navigation method for the autonomous underwater vehicle (AUV) *C-Ranger* applying information-filter-based simultaneous localization and mapping (SLAM), and its sea trial experiments in Tuandao Bay (Shangdong Province, P.R. China). Weak links in the information matrix in an extended information filter (EIF) can be pruned to achieve an efficient approach-sparse EIF algorithm (SEIF-SLAM). All the basic update formulae can be implemented in constant time irrespective of the size of the map; hence the computational complexity is significantly reduced. The mechanical scanning imaging sonar is chosen as the active sensing device for the underwater vehicle, and a compensation method based on feedback of the AUV pose is presented to overcome distortion of the acoustic images due to the vehicle motion. In order to verify the feasibility of the navigation methods proposed for the *C-Ranger*, a sea trial was conducted in Tuandao Bay. Experimental results and analysis show that the proposed navigation approach based on SEIF-SLAM improves the accuracy of the navigation compared with conventional method; moreover the algorithm has a low computational cost when compared with EKF-SLAM.

## 1. Introduction

Autonomous Underwater Vehicles (AUVs) have portable energy and self-control ability which make them different from remote operate vehicles (ROVs). They are suitable for commercial and military tasks underwater, under-ice or in other environments [1,2]. In the past few decades, the world plans to build or has built about 200 AUVs, such as the well-known REMUS by WHOI [3] or MIT Sea Grant’s Odyssey [4]. As the applications of AUVs are spreading to deeper seas and longer distances, high accuracy navigation capability will play a vital role. Inertial navigation systems (INS) are widely used in AUVs, but the navigation errors accumulate over time and waves and currents exacerbate this. Though the errors can be reduced periodically by using GPS, electromagnetic signals decay very quickly in the water, so the navigation of underwater vehicles cannot rely on GPS. As acoustic signals decay extremely slowly in seawater, acoustic navigation is widely used in three ways: long baseline, short baseline, ultra short baseline. However, deployment and recovery of the baseline is time-consuming and expensive, which limits acoustic navigation in large-scale environments [5]. In addition, it is very difficult to obtain prior maps of large-scale complex underwater environments. For all the reasons mentioned above, autonomous underwater navigation is considered one of the most challenging issues for AUVs [6].

Simultaneous localization and mapping (SLAM) does not require the aid of *a priori* information about the underwater environment, as when the vehicle moves in the water, its onboard sensors perceive the external environment and extract useful information, then form a map of the environment incrementally while positioning itself [7,8]. SLAM has attracted immense attention since the 1990s as the solution for true autonomous navigation [9], and a handful of algorithms have emerged for this purpose such as EKF-based SLAM, PF-based SLAM and IF-based SLAM. Using a parametric model, EKF-SLAM can be described as a posterior probability distribution parameterized by a state vector and covariance matrix. The algorithm consists of two main steps: prediction and update, which can be summarized as an iterative estimate and calibration process [10,11]. However, the map in EKF-SLAM comprises a covariance between the vehicle state and environmental features which must be processed in each estimate and correct step. As a result, It achieves *O*(*n*^2^) (the number of environmental features) computational complexity. To deal with the limitations, people divide the world into a handful of sub-maps; each containing *l* features [12–15]. Thus, computational complexity can be reduced to *O*(*l*^2^), but the tradeoff is sacrificing the convergence speed.

Particle filtering (PF) is different from KF, which solves SLAM problem by the particles based on the conditional independence. Fast SLAM, proposed by Montemerlo [16,17], is a very popular SLAM algorithm based upon particle filters. Fast SLAM decomposes SLAM into estimation of the robot path and estimate of the location of the features in the map. Once the vehicle path is established, the features are only related with the vehicle pose [18]. The particle filter is efficient in computation and can represent non-linear, non-Gaussian motion modes, but the more complex the environment is, the more particles are required to describe the posterior probability distribution. As a result, the algorithm complexity increases. In addition, the re-sampling will lead to loss of validation and diversity of particles which results in sample depletion.

Extended Information Filter (EIF) is the information form of EKF, parameterized by the information matrix and information vector corresponding to the EKF [19]. The time projection is efficient as it is quadratic in the number of measurements and not the size of the map. However, recovering the mean from the information matrix and information vector requires a costly *O*(*n*^3^), matrix inversion, so the computational complexity is even higher than for EKF. It is found that the vast majority of the information matrix elements are close to zero, while the information matrix is dominated by a small number of diagonal elements [20]. If the elements are made approximately close to zero, that is pruning weak links in the information matrix, an approximate representation which is the so-called Sparse Extended Information Filter (SEIF) proposed by Thrun *et al.* is obtained [21,22]. All the basic update formulae can be implemented in constant time, irrespective of the size of the map, which greatly improves computational complexity. There are a variety of new information filter-based algorithms such as TJFT [23], Treemap [24], ESEIF [25,26], but theoretical analysis and experimental evidence show that SEIF-SLAM is computationally efficient and consistent in the relative map, so the SEIF-SLAM algorithm is preferred in our work.

The *C-Ranger* AUV was developed in Underwater Vehicle Laboratory in Ocean University of China. It is equipped with a variety of onboard equipment for sensing vehicle pose and environment. In this paper we mainly address the autonomous navigation method for the *C-Ranger* AUV. To verify the advantages of SEIF-SLAM, the application of SEIF-SLAM in AUV navigation via sea trial experiments has been studied; also the data processing approach of sonar had been presented in this work. For the mechanical scanning imaging sonar chosen as the principal sensor for active sensing of undersea obstacles, a compensation method based on feedback of the AUV pose had been used to overcome distortion of the acoustic images due to the vehicle motion. Sea trials in Tuandao Bay were carried out to verify the feasibility of the proposed methods. The experimental results and analysis show that the proposed navigation approach based on SEIF-SLAM improved the accuracy of navigation when compared with conventional method; moreover the algorithm has a low computational cost when compared with EKF-SLAM.

The remainder of the paper is organized as follows: in the next section, the SEIF-SLAM algorithm for our *C-Ranger* AUV is presented, including the main processing steps. Section 3 describes the *C-Ranger* AUV and on-board sensors employed in the SLAM module. Further, sonar data processing is discussed in Section 4, including point-feature extraction and motion-distortion compensation. The sea trial experiments is described in Section 5, and the performance of the proposed navigation method is evaluated; then the results and possible future improvements are discussed in Section 6. Finally, we summarize the results and draw the fundamental conclusions.

## 2. A SEIF-SLAM Algorithm for the *C-Ranger* AUV

AUV travels undersea at a certain depth in most cases, so the bidimensional vehicle-landmark model was adopted to represent the AUV and landmarks (also called features) in the undersea environment. The vehicle pose **x***_t_* = (*x_t_*, *y_t_*, *θ_t_*)*^T^* and the set of map features **M** = (**m***_i_*, 1 ≤ *i* ≤ *N*)*^T^* are stored in the state vector:

(1) $$\xi_{t}={(x_{t}^{T},M^{T})}^{T}$$

**Figure 1.**
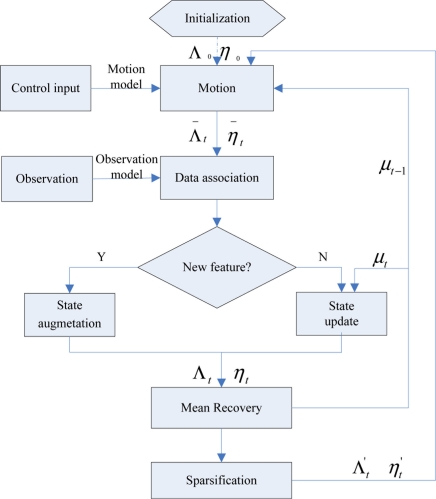
The flow chart of the SEIF-SLAM algorithm.

Like in Smith *et al.* [27], a first-order linearization of the motion and measurement models is employed considering the uncertainty in the data as independent, white Gaussian noise. Corresponding to the Gaussian distribution in Appendix, SEIF-SLAM can be presented as the following posterior probability distribution:

(2) $$p\left(x_{t},M|Z^{t},U^{t}\right)=N\left(\mu_{t},\Sigma_{t}\right)=N\left(\left[\begin{array}{l} \mu_{x_{t}}\\ \mu_{M} \end{array}\right],\left[\begin{array}{ll} \Sigma_{x_{t}X_{t}} & \Sigma_{x_{t}M}\\ \Sigma_{Mx_{t}} & \Sigma_{\mathrm{MM}} \end{array}\right]\right)=N^{-1}\left(\eta_{t},\Lambda_{t}\right)=N^{-1}\left(\left[\begin{array}{l} \eta_{x_{t}}\\ \eta_{M} \end{array}\right],\left[\begin{array}{ll} \Lambda_{x_{t}X_{t}} & \Lambda_{x_{t}M}\\ \Lambda_{Mx_{t}} & \Lambda_{\mathrm{MM}} \end{array}\right]\right)$$

where, **μ***_t_* is the mean of the state vector and **η***_t_* is the information vector, Σ*_t_* and Λ*_t_* denote the covariance and information matrix respectively, Z*^t^* and U*^t^* are the history of observational data and motion control inputs. To calculate the probability distribution, the algorithm mainly includes motion update, measurement update, sparsification, mean recovery and other steps. Figure 1 shows the structure of the algorithm.

### (A) Motion Update Step

The motion update step predicts the distribution over the new robot pose from time *t* − 1 to time *t* and subjects it to a Markov model, in general, a nonlinear function f of the previous pose **x**_*t*−1_ and the control inputs **u***_t_*. Equation (3) is the first-order linearization about the mean robot pose **μ**_*x*_*t*–1__, *F* denotes the Jacobian matrix about the state vector at time *t* − 1 and the term **v***_t_* ∼ N(0,Q*_t_*) represents the white Gaussian noise in the model, Q*_t_* is the noise covariance:

(3) $$x_{t}=f\left(x_{t-1},u_{t}\right)+v_{t}\approx f\left(\mu_{x_{t-1}},u_{t}\right)+F\left(x_{t-1}-\mu_{x_{t-1}}\right)+v_{t}$$

**Figure 2.**
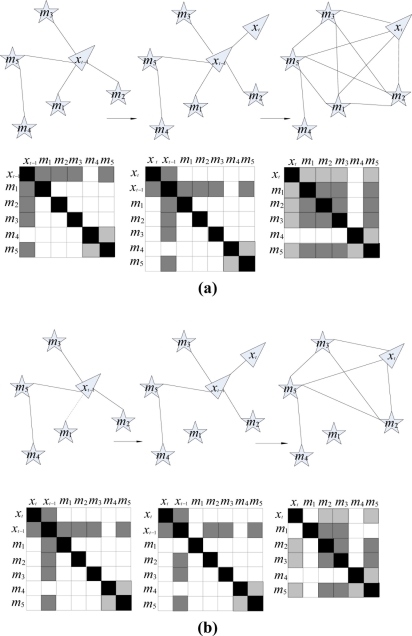
The evolution of the information matrix: **(a)** The evolution of the information matrix without a sparsification step. **(b)** The evolution of the information matrix when breaking weak links.

The motion update step can be implemented in two sub-steps. First, as in Figure 2(a), the state vector is grown to include the new robot pose ${\hat{\xi }}_{t}={\left(x_{t-1}^{T},x_{t}^{T},M^{T}\right)}^{T}$. According to the work by Eustice *et al.* [25,26], the augmentation of the information matrix and vector are given in Equations (4) and (5):

(4) $${\hat{\Lambda }}_{t}=\left[\frac{\left(\Lambda_{x_{t-1}x_{t-1}}+F^{T}Q_{t}^{-1}F\right)}{\begin{array}{l} -Q_{t}^{-1}F\\ \Lambda_{{\mathrm{Mx}}_{t-1}} \end{array}}|\frac{\begin{array}{cc} -F^{T}Q_{t}^{-1} & \Lambda_{x_{t-1}M} \end{array}}{\begin{array}{cc} Q_{t}^{-1} & 0\\ 0 & \Lambda_{\mathrm{MM}} \end{array}}\right]=\left[\frac{{\hat{\Lambda }}_{t}^{11}}{{\hat{\Lambda }}_{t}^{21}}|\frac{{\hat{\Lambda }}_{t}^{12}}{{\hat{\Lambda }}_{t}^{22}}\right]$$

(5) $${\hat{\eta }}_{t}=\left[\frac{\eta_{x_{t-1}}-F^{T}Q_{t}^{-1}\left(f\left(\mu_{x_{t-1}},u_{t}\right)-F\mu_{x_{t-1}}\right)}{\begin{array}{c} Q_{t}^{-1}\left(f\left(\mu_{x_{t-1}},u_{t}\right)-F\mu_{x_{t}}\right)\\ \eta_{M} \end{array}}\right]=\left[\frac{{\hat{\eta }}_{t}^{1}}{{\hat{\eta }}_{t}^{2}}\right]$$

Secondly, **x***_t_* is marginalized from the posterior probability distribution to achieve the desired distribution over ${\hat{\xi }}_{t}={\left(x_{t}^{T},M^{T}\right)}^{T}$ according to Appendix. The predicted information matrix Λ̄*_t_* and information vector **η̄***_t_* are:

(6) $${\bar{\Lambda }}_{t}={\hat{\Lambda }}_{t}^{22}-{\hat{\Lambda }}_{t}^{21}{\left({\hat{\Lambda }}_{t}^{11}\right)}^{-1}{\hat{\Lambda }}_{t}^{12}$$

(7) $${\bar{\eta }}_{t}={\hat{\eta }}_{t}^{2}-{\hat{\Lambda }}_{t}^{21}{\left({\hat{\Lambda }}_{t}^{11}\right)}^{-1}{\hat{\eta }}_{t}^{1}$$

### (B) Measurement Update Step

Sonar senses environment features actively in the experiment and observations of features are the key to reduce the uncertainty in the estimates for the robot pose and the map. The measurement step is also subject to a Markov model which is a nonlinear function h of the state vector **ξ***_t_*. Equation (8) shows the first-order linearization about the mean state vector **μ***_t_* and observed features with the Jacobian H evaluated at this mean. The term **w***_t_* ∼ *N* (0, R*_t_*) represents the white Gaussian noise in the model, *R_t_* is the noise covariance:

(8) $$z_{t}=h\left(\xi_{t}\right)+w_{t}\approx h\left(\mu_{t}\right)+H\left(\xi_{t}-\mu_{t}\right)+w_{t}$$

When a feature is observed repeatedly, it will be used to update the state estimation. According to [19] by Thrun *et al.*, the information matrix Λ*_t_* and the information vector **η***_t_* can be obtained:

(9) $$\Lambda_{t}={\bar{\Lambda }}_{t}+H^{T}R_{t}^{-1}H$$

(10) $${\bar{\eta }}_{t}={\bar{\eta }}_{t}+H^{T}R_{t}^{-1}\left(z_{t}-h\left(\mu_{t}\right)+H\mu_{t}\right)$$

with:

$$H=\left(\begin{array}{llllllll} \frac{\partial h}{{\partial x}_{t}} & 0 & \cdots & 0 & \frac{\partial h}{{\partial m}_{i}} & 0 & \cdots & 0 \end{array}\right)$$

The observation model is only a function of the vehicle state **x***_t_* and the observed features **m***_i_* (1 ≤ *i* ≤ *N*) at time *t*, so H is zero everywhere except at positions associated with **x***_t_*, **m***_i_*. The measurement update step only strengthens the links between vehicle pose and features to be updated, so the sparseness of information matrix can never be changed.

### (C) Sparsification Step

To make information sparse, SEIF’s strategy for sparsification is based on partitioning the map into a union of three disjoint sets:

$$M=\left\{M^{+},M^{-},M^{0}\right\}$$

where **M**^+^ is the current active features that will remain active after sparsifying, **M**^−^ denotes the passive features that will remain passive, **M**^0^ comprises the active features that will be made passive [28]. So the SLAM posterior can be factored into:

(11) $$\begin{array}{ll} p\left(x_{t},M|Z^{t},U^{t}\right) & =p\left(x_{t},M^{+},M^{-},M^{0}|Z^{t},U^{t}\right)\\ & =p\left(x_{t},|M^{+},M^{-},M^{0},Z^{t},U^{t}\right)p\left(M^{+},M^{-},M^{0}|Z^{t},U^{t}\right) \end{array}$$

Due to the conditional independence between the pose and **M**^−^ of the vehicle, hence we can set **M**^−^ to an arbitrary value. Here, it is simply chosen as 0. The evolution of information matrix is shown in Figure 2.

Figure 2(a) illustrates the situation before sparsification: according to the partition, **M**^+^ = {**m**_1_, **m**_2_, **m**_3_, **m**_4_}, **M**^−^ = {**m**_4_} at time *t*, the information matrix tends to be dense after time-projection step (state augmentation and marginalization) which is the main reason for creating weak links.

By eliminating the weak links between the features and vehicle pose in the information matrix, a sparse approximation that allows for efficient SLAM can be achieved. As shown in Figure 2(b), **m**_1_ is made passive before state augmentation and marginalization. It is obvious that the information matrix is sparse. To sparsify the information matrix, the posterior is approximated by dropping the dependence on **M**^0^ in the first term of Equation (11):

(12) $$\begin{array}{ll} p\left(x_{t},M|Z^{t},U^{t}\right) & \approx p\left(x_{t}|M^{+},M^{-}=0,Z^{t},U^{t}\right)p\left(M^{+},M^{-},M^{0}|Z^{t},U^{t}\right)\\ & =\frac{p\left(x_{t},M^{+}|M^{-}=0,Z^{t},U^{t}\right)}{p\left(M^{+}|M^{-}=0,Z^{t},U^{t}\right)}p\left(M^{+},M^{-},M^{0}|Z^{t},U^{t}\right) \end{array}$$

The information matrix for the distribution *p*(**x***_t_*, **M**^+^, **M**^0^ | **M**^−^ = 0) is:

(13) $${\tilde{\Lambda }}_{t}=S_{x,M^{+},M^{0}}S_{x,M^{+},M^{0}}^{T}\Lambda_{t}S_{x,M^{+},M^{0}}S_{x,M^{+},M^{0}}^{T}$$

where S_*x*,*M*^+^, *M*^0^_ denotes a projection matrix, used to extract the submatrix of all state variables except **M**^0^. Let $\Lambda_{t}^{1}$, $\Lambda_{t}^{2}$, $\Lambda_{t}^{3}$ denote the information matrices for the terms *p*(**x***_t_*, **M**^+^ | **M**^−^ = 0, Z*^t^*, U*^t^*), *p*(**M**^+^ | **M**^−^ = 0, Z*^t^*, U*^t^*) and *p*(**M**^+^, **M**^−^, **M**^0^ | Z*^t^*, U*^t^*) respectively, so the sparse information matrix can be obtained as Equation (14) by putting these matrices together according to Equation (12):

(14) $$\begin{array}{lll} {\Lambda^{\prime }}_{t} & = & \Lambda_{t}^{1}-\Lambda_{t}^{2}+\Lambda_{t}^{3}\\ & = & \Lambda_{t}-{\tilde{\Lambda }}_{t}S_{M^{0}}{\left(S_{M^{0}}^{T}{\tilde{\Lambda }}_{t}S_{M^{0}}\right)}^{-1}S_{M^{0}}^{T}{\tilde{\Lambda }}_{t}\\ & & +{\tilde{\Lambda }}_{t}S_{x,M^{0}}{\left(S_{x,M^{0}}^{T}{\tilde{\Lambda }}_{t}S_{x,M^{0}}\right)}^{-1}S_{x,M^{0}}^{T}{\tilde{\Lambda }}_{t}-\Lambda_{t}S_{x}{\left(S_{x}^{T}\Lambda_{t}S_{x}\right)}^{-1}S_{x}^{T}\Lambda_{t} \end{array}$$

The resulting information vector is now obtained by the following simple consideration:

(15) $${\eta^{\prime }}_{t}={\Lambda^{\prime }}_{t}\mu_{t}=\left(\Lambda_{t}-\Lambda_{t}+{\Lambda^{\prime }}_{t}\right)\mu_{t}=\eta_{t}+\left({\Lambda^{\prime }}_{t}-\Lambda_{t}\right)\mu_{t}$$

### (D) Mean Recovery

The information form of the Gaussian is parameterized by its information vector and information matrix. However, the linearization, sparsification, measurement update steps require the mean of the state vector. According to the duality of Gaussian distribution in Appendix, we can get the mean vector:

(16) $$\mu_{t}=\Lambda_{t}^{-1}\eta_{t}$$

Due to the great cost of full state recovery and the steps that require the mean of state vector oftentimes only require a subset of the state mean, the mean vector can be recovered by dividing it into two sets:

(17) $$\left[\begin{array}{cc} \Lambda_{\mathrm{ll}} & \Lambda_{\mathrm{lb}}\\ \Lambda_{\mathrm{bl}} & \Lambda_{\mathrm{bb}} \end{array}\right]\left[\begin{array}{c} \mu_{l}\\ \mu_{b} \end{array}\right]=\left[\begin{array}{c} \eta_{l}\\ \eta_{b} \end{array}\right]$$

where **μ***_l_* is the “local portion” that needs to be recovered and **μ***_b_* is the “begin portion” of the map. If current estimation for **μ***_b_* is kept fixed, an estimate of **μ***_l_* can be obtained as:

(18) $${\hat{\mu }}_{l}=\Lambda_{\mathrm{ll}}^{-1}\left(\eta_{l}-\Lambda_{\mathrm{lb}}{\hat{\mu }}_{b}\right)$$

## 3. The *C-Ranger* AUV and On-Board Sensors

### 3.1. C-Ranger AUV

**Figure 3.**
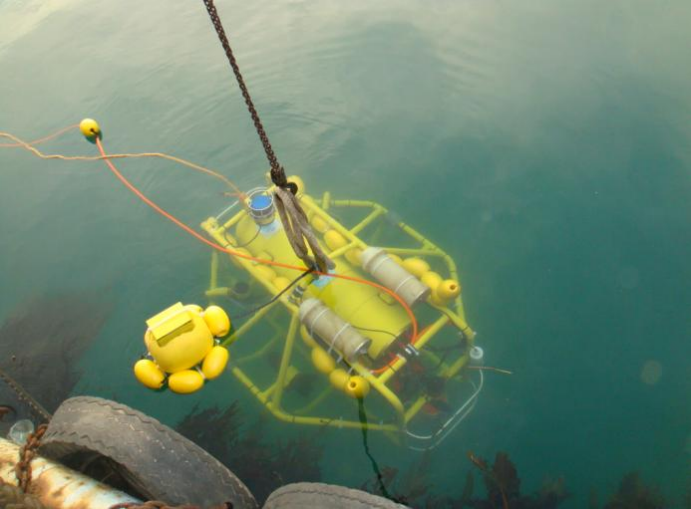
*C-Ranger* in deployment.

**Figure 4.**
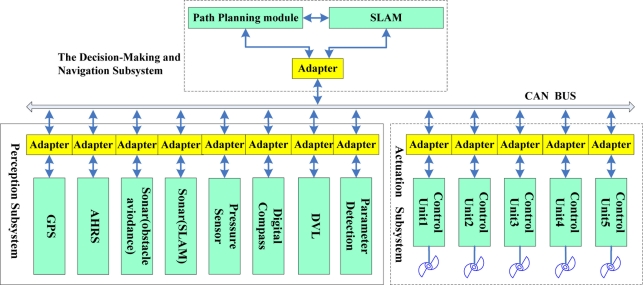
Control architecture of the *C-Ranger*.

The *C-Ranger* is an open-frame AUV with dimensions of 1.6 m × 1.3 m × 1.1 m (length, width and height), as shown in Figure 3. There are two electronic cabins and five underwater propeller thrusters. The control architecture of *C-Ranger* is illustrated in Figure 4. The AUV has good maneuverability due to its five DOFs, including surge, heave, roll, pitch, and yaw. The thrust system of this platform consists of five propeller thrusters, where two thrusters are parallel to the bow direction, and installed in the abdomen to provide horizontal thrust for mainly controlling the surge and roll, The other three thrusters are employed to provide vertical thrust to control the heave, roll, and pitch, two of which are installed on both sides of the bow, and the remaining one is installed on the rear of the vehicle. The upper hull of the *C-Ranger* is the instrument compartment housing sensors, two industrial computers, a communication module, internal monitoring module and other equipment, while the lower hull is the power and thrust system composed of lithium-ion batteries, power management module, motor-driver module, *etc*. The maximum speed of the *C-Ranger* is 3 knots, and it can operate for up to 8 hours when fully charged (tested at speed of one knot).

### 3.2. On-Board Sensors

A number of sensors are installed on the *C-Ranger*, some of them are explicitly related to SLAM. These sensors are basically divided into two groups: the internal and the external. Internal sensors include digital compass, gyro, Attitude and Heading Reference System (AHRS) and pressure sensor. External sensors include mechanical scanning sonar, Doppler Velocity Log (DVL), altimeter, CCD camera and GPS.

### (A) Mapping-Related Sensor: Active Imaging Sensor

A mechanical-scanning forward-looking sonar (Super Seaking DST, Tritech) used for active sensing of environment features is installed at the front top of *C-Ranger*. It is the principal sensor of the *C-Ranger* AUV. The operating frequency of the sonar is 675 kHz, and its maximum range is 300 meters. Generally the scanning rate of this kind of sonar is slow, which would make the acoustic image distorted. A compensation for motion-induced distortion will be addressed in the next section.

### (B) Velocity Sensor: DVL

The DVL (NavQuest600, LinkQuest) is used to provide the velocities of the vehicle relative to the seabed. In addition, the NavQuest600 can provide other information: pitch angle, roll angle, heading, altitude, depth, temperature and velocities relative to the ocean currents.

### (C) Angular Sensors: AHRS and Gyro

The AHRS (M2, Innalabs) is used to produce attitude information, and the gyro (VG951D) is used to measure angular velocity in the process of AUV navigation. AHRS M2 is a low-cost high-performance inertial navigation system, and magnetic interference will not affect the accuracy of headings over short times.

### (D) Positioning Sensor: GPS

To evaluate navigation performance of the *C-Ranger*, a high-precision and high-dynamic GPS receiver is employed. In the absence of SA, the positioning accuracy is up to 1.1 m (CEP), and the data update rate is up to 20 Hz. The GPS sensor can offer a benchmark to evaluate the estimation of robot trajectory.

## 4. Data Processing of Sonar

Raw data measured by sensors should be pretreated before it is used in the navigation algorithm. The amount of the raw data from most sensors is not very high, so they can be used in SLAM simply after denoising and synchronizing. Sonar is very essential for the environmental map building, but the large amount of sonar data constrains the real-time navigation. Furthermore, its long scan-cycle will lead to motion-distortion. For the reasons, it is vital to handle sonar data appropriately before being used in SLAM algorithm, thus the data processing of sonar is presented in this section.

### 4.1. Feature Extraction of Sonar Data

Currently methods based on image processing are popular ones applied to extract features from raw sonar data [29,30], but they are generally too slow for the applications such as AUV navigation using mechanical-scanning-sonar. A real-time data processing is proposed in the next paragraphs.

**Figure 5.**
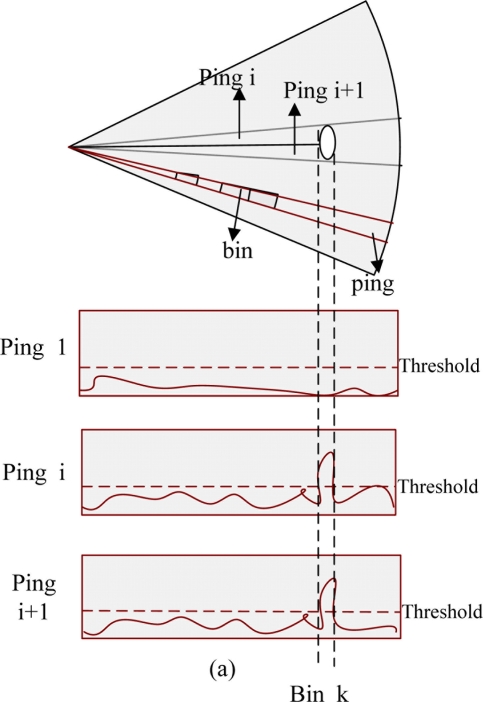
The schematic diagram of sonar scanning obstacles.

Sonar transmits sound waves stepwisely and receives them after encountering obstacles; we call each sound wave beam one *ping* and every *ping* can be divided into several bins. The relative position of a bin reflects the distance between the sonar and the obstacle, the larger the intensity of bins are, the more obvious features there are, and *vice versa* [31]. As Figure 5 shows, sonar scans stepwisely in a given sector, and finds one obstacle in the *k* – *th* bin of the *i* – *th* and (*i* + 1) – *th ping*, respectively. Then the intensity and the relative position for every bin whose intensity is beyond a predefined threshold will be translated into corresponding feature information.

**Figure 6.**
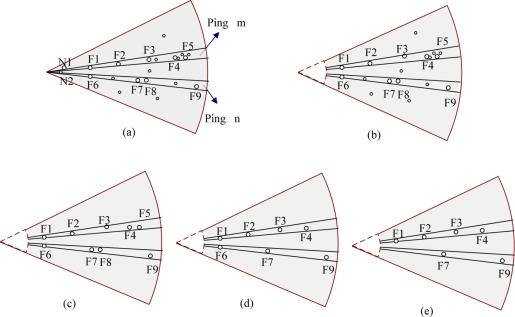
The schematic diagram of sonar data sparsification.

Noise in raw data should be eliminated, and the number of features extracted should be as low as possible due to the demands of real-time navigation, in other words, we need to make the features sparse, Figure 6(a–e) show the process. In Figure 6(a) the sonar observes *N*_1_, *N*_2_ which are very close to the sonar head (transducer), high-intensity features in two *pings*, *i.e.*, *ping : m* = {*F*_1_, *F*_2_, *F*_3_, *F*_4_, *F*_5_}, *ping : n* = {*F*_6_, *F*_7_, *F*_8_, *F*_9_}, as well as any low-intensity features (not labeled in the figure). First, we eliminate the noise caused by the sonar itself: according to the distance relationship between sonar and the frame of AUV, we remove the noise close to the vehicle as Figure 6(b) shows. The second step is to eliminate background noise: removing features whose intensity is below the predefined threshold, we will retain features *F*_1_∼*F*_9_ with high intensity in Figure 6(c). The third step is the processing of a single-*ping*: selecting local *prominent* points in each *ping* in order to ensure that the target-related information is retained, then removing redundant features near the *prominent* point by using a distance threshold. Thus we will get features in each *ping* and effectively ensure that one target will not match more than one feature. In *ping* m, F5 is within the sparsification threshold (a distance value) of the F4, so are F8 and F7 in *ping* n. Then because of F4 > F5, F7 > F8 in intensity, we will get the results shown in Figure 6(d).

Generally the retained features are still too dense, even after the above radial processing, so we need to conduct further sparsification between *ping*s. The procedure is similar to the above step 3, whether one feature is retained or not depends on a threshold as well, but such operations are along direction of a circular arc. The results shown in Figure 6(e) can be obtained in this way. The above processing actually can cut off most redundant information, and greatly reduce the number of features without affecting positioning accuracy. The thresholds mentioned above depend on several factors, such as the distribution of environmental features and accuracy of features’ positions, moreover, the requirement by efficiency of SLAM is also taken into account if necessary.

### 4.2. Compensation on Motion-Induced Distortion for Mechanical-Scanning Sonar

**Figure 7.**
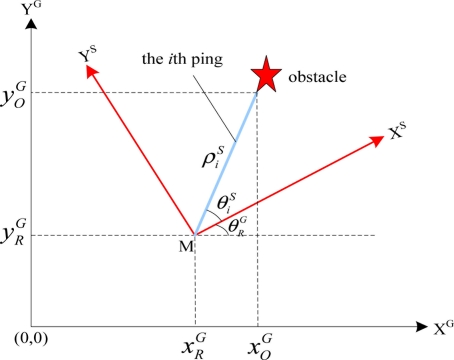
Compensation of the motion-induced distortion.

The transducer head of a mechanical-scanning imaging sonar usually needs a considerable period of time to perform a 360° rotation. This is an important issue that has to be taken into account when operating with such sonar mounted on an AUV, since the resulting acoustic images can get distorted as a consequence of the vehicle’s motion. Generally, this effect can be ignored for low velocities. For higher velocities, it has to be specially dealt with. In the case of the *C-Ranger*, we use position feedback to undistort the data. The principle of data undistortion is shown in Figure 7, where, superscripts ‘G’ and ‘S’ stand for the global coordinate frame and the sonar coordinate frame respectively; subscripts ‘R’ and ‘O’ denote variables for the vehicle and the features, respectively.

Firstly, a global coordinate frame is built at the starting time of every circle of sonar scanning. In this global coordinate frame, the vehicle’s initial position is supposed at the origin (0, 0). Given the vehicle moves to point M after the scan interval *t* for one *Ping*. During this period, the vehicle’s velocities *v_x_* and *v_v_* and can offered by SLAM module, and the vehicle’s displacement ( $x_{R}^{G}$, $y_{R}^{G}$) can be obtained as:

(19) $$\{\begin{array}{c} x_{R}^{G}=\nu_{x}\cdot t\\ y_{R}^{G}=\nu_{y}\cdot t \end{array}$$

Simultaneously, the vehicle’s rotation angle $\theta_{R}^{G}$ can be provided by outputs of the SLAM module. Then, in the vehicle coordinate frame, the feature (marked as pentagram in Figure 7) is detected with a distance $\rho_{i}^{s}$ from the vehicle (*i.e.*, sonar) and the angle $\theta_{i}^{S}$, where *i* stands for index of the *Bin* in the *Ping*. Finally, we need to transform the polar coordinates ( $\rho_{i}^{s}$, $\theta_{i}^{S}$) to the Cartesian coordinates in the global coordinate frame and incorporate the motion displacement of the vehicle, thus the compensation formula can be presented as:

(20) $$\{\begin{array}{c} x_{O}^{G}(i)=x_{R}^{G}+\rho_{i}^{S}•\text{sin}\left(\theta_{i}^{S}+\theta_{R}^{G}\right)\\ y_{O}^{G}(i)=y_{R}^{G}+\rho_{i}^{S}•\text{cos}\left(\theta_{i}^{S}+\theta_{R}^{G}\right) \end{array}$$

By parity of reasoning, after the acquisition of every scanning *Ping*, the position of features will be corrected according to the above formula. Once the sonar completes one circle of scan, the undistorted image will be obtained without doubt.

**Figure 8.**
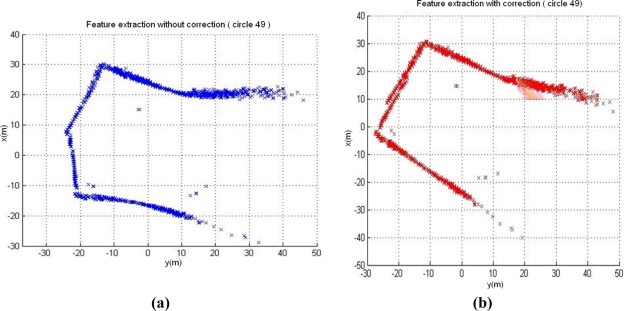

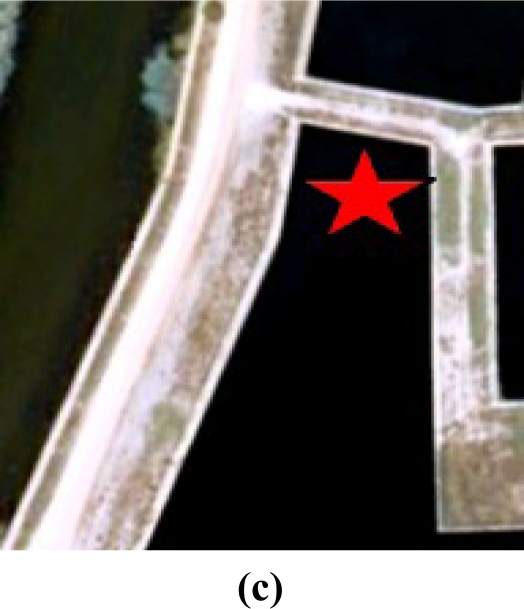
Effect of the vehicle motion on acoustic images. **(a)** Raw sonar image. **(b)** Corrected sonar image. **(c)** Zenithal view of the Abandoned Marina [32].

We use the data in the 49th circle of the Abandoned Marina Dataset [32] to verify the effect of this correction method. Figure 8(a) shows the acoustic image built from the raw sonar data. Since the vehicle’s motion has been ignored during the generation of the image, obvious distortion of the observed features appears when comparing it with the aerial image of the test scenario in Figure 8(c).

The corrected image using the proposed method is shown in Figure 8(b). Obviously the distortion of the image is almost cancelled and a more accurate image is represented. The result demonstrates that our correction approach is effectual.

## 5. Experiment and Results Analysis

### 5.1. Experiment in Tuandao Bay

**Figure 9.**
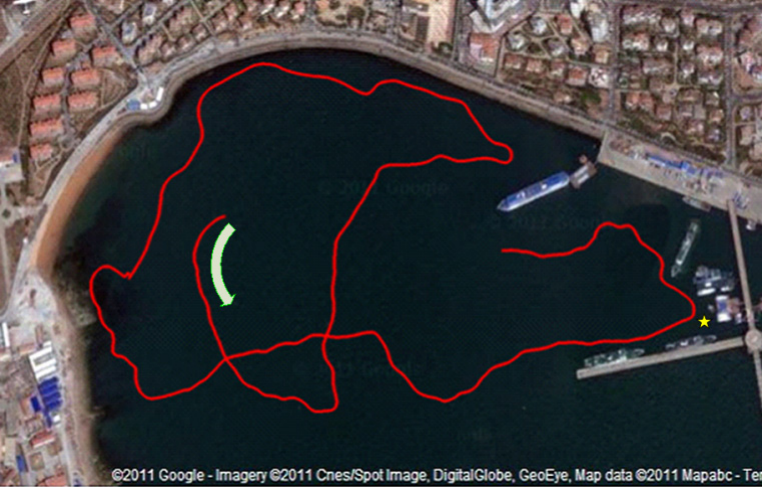
The satellite map of Tuandao Bay and the trajectory of the *C-Ranger* by GPS.

The experiment was performed at Tuandao Bay in Qingdao (China). The satellite map (from Google Earth) and AUV trajectory measured by GPS are shown in Figure 9, where a starting point with direction is marked using a green arrow. The total travel in the experiment is up to 2,812 m with the sailing speed about 1 knot. The scan sector of the principal sonar was configured to 180° with a scanning range of 100 meters.

### 5.2. Experimental Results

**Figure 10.**
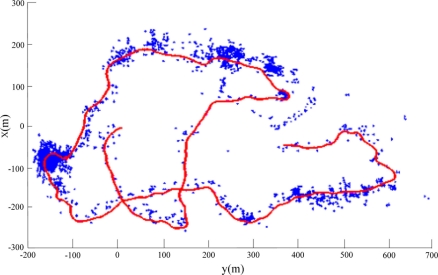
The features extraction without sparsification.

**Figure 11.**
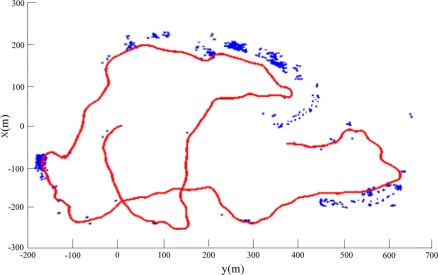
The features extraction with sparsification.

In the experiment, the global coordinates measured by a GPS receiver in the *C-Ranger*, with a GPS antenna in a buoy connected to the AUV via a short cable, will be taken as the *ground truth* used for the evaluation of SLAM results. Figure 10 shows the features (marked with blue asterisk) extracted directly from sonar data without using sparsificaton. The point-features in the picture are very dense, and there are many “features” away from the possible objects such as vessels and sea bank, actually a large part of them are noise. As known, the large amount of point-features would increase the computational cost of SLAM and limit the real-time performance of AUV navigation. So the approach presented in Section 4 is necessary to eliminate redundant ones and reduce the number of features. Figure 11 illustrates the result after denoising and sparsifying, the remained features are basically the most representative ones needed to describe the undersea objects.

**Figure 12.**
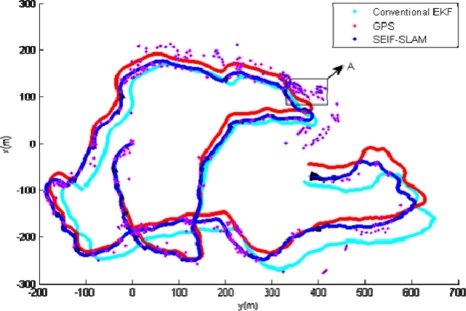
Comparison of the trajectories for GPS (red line), EKF (light blue line) and the SEIF-SLAM algorithm.

A comparison of the trajectories for GPS (the red line), EKF (the light blue line) and the SEIF-SLAM algorithm (the blue line) as well as the features (blue points) is shown in Figure 12. Obviously, the deviation of SLAM relative to GPS is smaller than that of EKF, and the deviation of EKF tends to increase gradually. On the other hand, the location of the features obtained by SLAM approach match the actual environment landmarks quite well.

**Figure 13.**
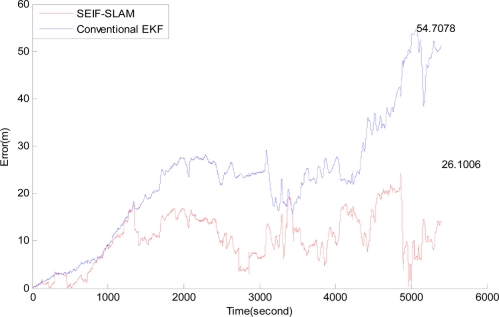
Plots of the error of conventional extended-Kalman-filter and SEIF-SLAM relative to GPS. The GPS data has been used as ground truth.

Figure 13 presents the estimation errors of SEIF-SLAM (the red line) and EKF (the blue line) relative to GPS, respectively. In this figure we can see that the error of SEIF-SLAM is always smaller than that of EKF for the whole experiment except for a few tens of seconds in the beginning. The maximum error of SEIF-SLAM is 26.1 m which is only about 7.3‰ of the whole course, and appears at the time of 4,800 seconds after starting off. As shown in Figure 9, the corresponding point at this time is at the position marked as a yellow star. That is because there were many moving targets present such as little boats during the experiment which would impact the sonar observations.

**Figure 14:**
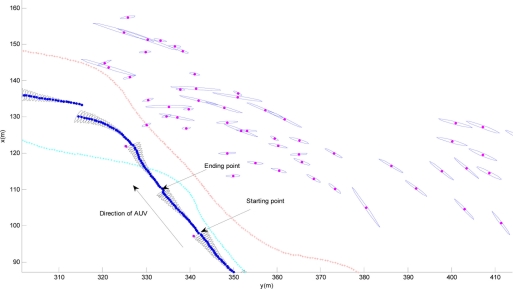
The uncertainty of vehicle pose and environment features (area A).

For more clarity, area A marked in Figure 12 is zoomed, as shown in Figure 14, in which the black and blue ellipses denote the uncertainty of vehicle pose and features separately. It is obvious from Figure 14 that the vehicle’s uncertainty increases for some time and then becomes very small suddenly. In fact, we note that the change of the uncertainty is cyclical: at the starting stage, the uncertainty for the pose is small and will increase gradually in one sonar-scanning-cycle because of error accumulation; at the ending point (*i.e.*, the end of one sonar-scanning-cycle), the pose estimation uncertainty becomes small because of the update step in the SEIF-SLAM algorithm. On the other hand, due to the sonar scanning noise, the features uncertainty in Figure 14 seems to not follow any rules. In fact, when some of the features are revisited, their uncertainty will become gradually smaller relative to other features. In general, the more accurate the pose estimation is and the more times the feature is re-observed, then the smaller the feature uncertainty is.

**Figure 15.**
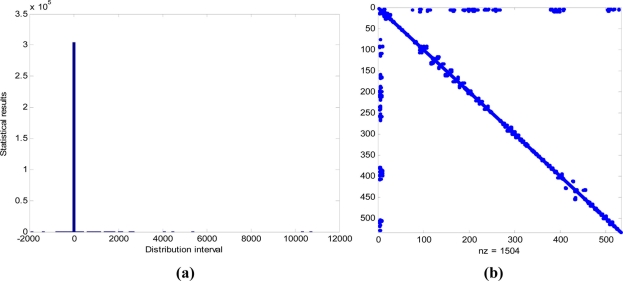
The statistical results of information matrix. **(a)** Statistical result of the elements magnitude of the information matrix; **(b)** Location of elements in the information matrix.

The sparsity of information matrix is the key to the lower computational complexity of SEIF-SLAM. Figure 15 shows the statistical results of the information matrix elements.

Figure 15(a) is the statistical result of the elements magnitude of the information matrix. We can find that the overwhelming majority of information matrix elements is close to zero while only a small number of them are far from zero. Figure 15(b) shows where these elements locate in the information matrix and the blue dot represents the non-zero elements which mainly locate in the diagonal and the two ends of the counter-diagonal of the information matrix. The diagonal elements are the respective variance of the pose and features while the counter-diagonal is the covariance of the last observed features, the links of which are not yet marginalized and approximated to zero.

**Figure 16.**
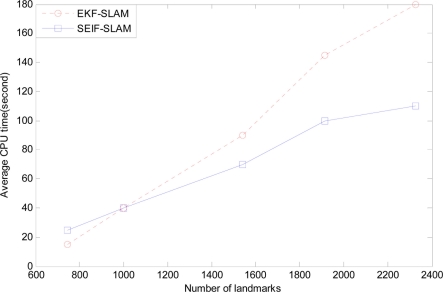
The comparison of average CPU time between SEIF-SLAM and EKF-SLAM.

**Figure 17.**
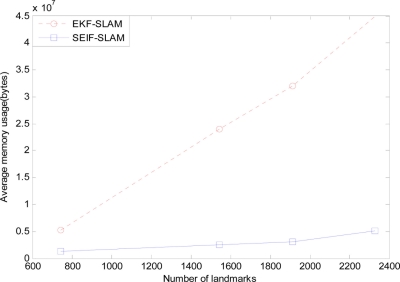
The comparison of average memory usage between SEIF-SLAM and EKF-SLAM.

To compare the performance of computational efficiency, 50 Monte Carlo experiments are carried out. Figures 16 and 17 present the comparisons of average CPU time and memory usage between EKF-SLAM and SEIF-SLAM.

As we can see that SEIF-SLAM is less efficient than EKF-SLAM when there are less than 1,000 features in the map. The reason for this is that when there are only few features, the impact brought by computation in sparsification step is much greater than that brought by the sparsity property. When the number of features is more than 1,000, SEIF-SLAM is more efficient than EKF-SLAM. Figure 17 demonstrates the difference between SEIF-SLAM and EKF-SLAM in storage. We can conclude that SEIF-SLAM need much less storage than that of EKF-SLAM and the gap increases when the number of features increases. In fact, it is because that SEIF-SLAM maintains information matrix which is sparse, which is far superior to the non-sparse matrix in storage.

## 6. Discussion

Like EKF-SLAM, the consistency of the SEIF-SLAM algorithm is not perfect. There are two reasons for this: on the one hand, error accumulation caused by nonlinear model linearization will easily result in inconsistency, and this problem is the same as the EKF algorithm and exists in most of the SLAM algorithms based on the Gaussian linear filters; On the other hand, the sparsification, which marginalized out the weak links (*i.e.*, the links between vehicle pose and further features), could affect consistency though it has been proved that SEIF-SLAM has relatively good consistency [26,28]. The ESEIF-SLAM algorithm [26] improves the consistency through the modification of the sparsification steps of the SEIF algorithm, but increases a little computation. The more efforts will be made to improve the consistency and reduce computational cost of the information-filter-based SLAM algorithm in our future work.

Secondly, the mechanical-scanning sonar used on the *C-Ranger* usually has a quite low scanning rate, it needs about 27 seconds to scan a 360° sector. As a result, SEIF-SLAM filter updates happen approximately 13.5 meters apart, and the frequency of measurement update is very slow. Obviously the main reason is the usage of the mechanical sonar. Therefore, the mechanical sonar will be exchanged for a multi-beam sonar to obtain a high update frequency on the next generation platform. High update frequency should be helpful to improve the accuracy of navigation for AUV a lot.

## 7. Conclusions

In this paper, the autonomous navigation method for the *C-Ranger* AUV had been addressed. To verify the advantages of SEIF-SLAM, the application of SEIF-SLAM for AUV navigation had been studied, the approach for data processing of sonar had also been presented in this work. The mechanical scanning imaging sonar is chosen as the principal sensor for active sensing of the undersea obstacles, a compensation method based on feedback of the AUV pose has been proposed to overcome distortions of the acoustic images due to the vehicle motion. Sea trial experiments in Tuandao Bay have been conducted to verify the feasibility of the proposed navigation methods for the *C-Ranger*. The experimental results and analysis demonstrated that the proposed navigation approach based on SEIF-SLAM improved the accuracy of navigation compared with conventional methods; moreover the algorithm achieves low computational cost when compared with EKF-SLAM.

## Appendix

### The Duality of Gaussian Distribution

Let **ξ***_t_* be a multi-dimensional random variable which subjects to Gaussian distribution *p*(**ξ***_t_*) ∼ *N*(**μ***_t_*, Σ*_t_*), where **μ***_t_* and Σ*_t_* denote the mean and covariance. This representation is often called the covariance form of Gaussian distribution. Expanding this representation in the exponential form, the following equivalent representation can be reached:

(A1) $$\begin{array}{ll} p\left(\xi_{t}\right) & =N\left(\mu_{t},\Sigma_{t}\right)\\ & =\frac{1}{\sqrt{\left|2\pi \Sigma_{t}\right|}}\text{exp}\left\{-\frac{1}{2}{\left(\xi_{t}-\mu_{t}\right)}^{T}\Sigma_{t}^{-1}\left(\xi_{t}-\mu_{t}\right)\right\}\propto \text{exp}\left\{-\frac{1}{2}{\left(\xi_{t}-\mu_{t}\right)}^{T}\Sigma_{t}^{-1}\left(\xi_{t}-\mu_{t}\right)\right\}\\ & =\text{exp}\left\{-\frac{1}{2}\left(\xi_{t}^{T}\Sigma_{t}^{-1}\xi_{t}-{2\mu }_{t}^{T}\Sigma_{t}^{-1}\xi_{t}+\mu_{t}^{T}\Sigma_{t}^{-1}\mu_{t}\right)\right\}\propto \text{exp}\left\{-\frac{1}{2}\xi_{t}^{T}\Sigma_{t}^{-1}\xi_{t}+\mu_{t}^{T}\Sigma_{t}^{-1}\xi_{t}\right\}\\ & =\text{exp}\left\{-\frac{1}{2}\xi_{t}^{T}\Lambda_{t}\xi_{t}+\eta_{t}^{T}\xi_{t}\right\}\propto N^{-1}\left(\eta_{t},\Lambda_{t}\right) \end{array}$$

The representation *p*(**ξ***_t_*) ∝ *N*^−1^ (**η***_t_*, Λ*_t_*) is the named information form of Gaussian distribution, and is parameterized by the information matrix Λ*_t_* and information vector **η***_t_*. The equivalence of the above two forms is called the duality of Gaussian distribution, that is:

(A2) $$\begin{array}{l} \;\;\Lambda_{t}=\Sigma_{t}^{-1},\eta_{t}=\Sigma_{t}^{-1}\mu_{t}\\ \mathrm{or}\\ \;\;\Sigma_{t}=\Lambda_{t}^{-1},\mu =\Lambda_{t}^{-1}\eta \end{array}$$

Equation (A3) shows these two representations about the random variable **ξ***_t_* that comprises two sub-vector **α** and **β**. The mean, covariance, information matrix and information vector are partitioned into their corresponding parts:

(A3) $$p\left(\xi_{t}\right)=p\left(\alpha ,\beta \right)=N\left(\left[\begin{array}{l} \mu_{\alpha }\\ \mu_{\beta } \end{array}\right],\left[\begin{array}{cc} \Sigma_{\alpha \alpha } & \Sigma_{\alpha \beta }\\ \Sigma_{\beta \alpha } & \Sigma_{\beta \beta } \end{array}\right]\right)=N^{-1}\left(\left[\begin{array}{l} \eta_{\alpha }\\ \eta_{\beta } \end{array}\right],\left[\begin{array}{cc} \Lambda_{\alpha \alpha } & \Lambda_{\alpha \beta }\\ \Lambda_{\beta \alpha } & \Lambda_{\beta \beta } \end{array}\right]\right)$$

It is noteworthy that these two equivalent representations lead to very different computational characteristics with respect to calculating the probability of marginalization and conditioning. Table A1 summarizes this difference where we can see the marginalization is relatively easy in covariance form while hard in information form and the opposite relation holds true for conditioning.

**Table A1.** The duality of Gaussian distribution.

	Marginalization *p*(**α**) = ∫ *p*(**α**, **β**)*d***β**	Conditioning *p*(**α**|**β**) = *p*(**α**, **β**)/*p*(**β**)
Covariance Form	**μ** = **μ***_α_*	$\mu^{\prime }=\mu_{\alpha }+\Sigma_{\alpha \beta }\Sigma_{\beta \beta }^{-1}\left(\beta -\mu_{\beta }\right)$
	Σ = Σ*_αα_*	$\Sigma^{\prime }=\Sigma_{\alpha \alpha }-\Sigma_{\alpha \alpha }\Sigma_{\beta \beta }^{-1}\Sigma_{\beta \alpha }$
Information Form	$\eta =\eta_{\alpha }-\Lambda_{\alpha \beta }\Lambda_{\beta \beta }^{-1}\eta_{\beta }$	**η**′ = **η***_α_* – Λ*_αβ_***β**
	$\Lambda =\Lambda_{\alpha \alpha }\Lambda_{\beta \beta }^{-1}\Lambda_{\beta \alpha }$	Λ′ = Λ*_αα_*
